# "Well, it's nobody's responsibility but my own." A qualitative study to explore views about the determinants of health and prevention of knee pain in older adults

**DOI:** 10.1186/1471-2458-10-148

**Published:** 2010-03-22

**Authors:** Clare Jinks, Bie Nio Ong, Tracey O'Neill

**Affiliations:** 1 Arthritis Research Campaign National Primary Care Centre, Primary Care Sciences, Keele University, Keele ST5 5BG, UK; 2 UKCRC Centre of Excellence for Public Health (NI), School of Medicine, Dentistry and Biomedical Sciences, Mullhouse Building, Grosvenor Rd, Belfast BT 12 6BJ, UK

## Abstract

**Background:**

Dahlgren and Whitehead's 'rainbow' outlines key determinants of health and has been widely adopted within public health policy and research. Public understanding regarding the determinants of health is, however, relatively unknown, particularly in relation to common chronic joint problems like knee pain. We aimed to explore individual attitudes to the prevention of knee pain, and assess how people make sense of their lives by using the rainbow model to explore social determinants of health.

**Methods:**

Twenty-eight semi-structured interviews were undertaken with older adults living in the community. The format of the interview enabled individuals to first tell their story, then the rainbow picture was used to further prompt discussion. Interviews were digitally recorded and transcripts were fully transcribed. Qualitative computer software package NVivo 2 was used to manage the data. Thematic analysis was undertaken.

**Results:**

Individual responsibility for health was a dominant theme although the role of health and statutory services was also recognised. Barriers to uptake of prevention activities included cultural perceptions, attitudes towards work and perceived costs of prevention activities. Participants used the rainbow for locating their personal life within a wider social, economic and policy context.

**Conclusions:**

People view individual responsibility as key to maintaining health and draw upon the past, present and future expectations when considering social determinants of their health. The rainbow picture does have relevance at the individual level and can help to formulate more dynamic and contextualised approaches to the prevention of health conditions in community living adults.

## Background

The thrust of English health policy on prevention remains dominated by a focus on major diseases, as reflected in the formulation and implementation of National Service Frameworks, and on changing individual behaviour and lifestyle. The latter is exemplified by the promotion of self-management programmes, initiatives such as Health Trainers and Health Literacy and the over-arching emphasis on choice (e.g. 'Choosing Health') [1]. The more recent government paper "Healthy Weight, Healthy lives: A Cross-Government Strategy for England" [2] outlines a more comprehensive approach, bringing together socio-economic and psychological factors with public services. Yet, the focal point remains the individual and his/her lifestyle which gives rise to some fundamental questions as to how people themselves perceive health and illness, and whether attempts to change lifestyles find resonance with how they actually live their lives.

**Figure 1:**
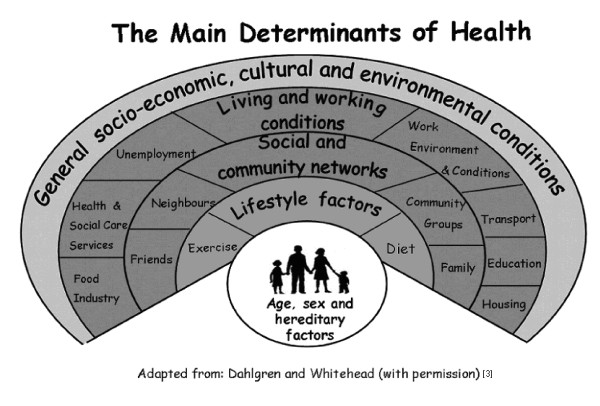
**Picture aid used in qualitative interviews with people to discuss prevention of knee pain and disability**.

The perspective presented by public health research as to how individual health is shaped starts from a different premise. Over the last two decades a number of models have been formulated that aim to explain the complex and multidimensional pathways to health, and inequalities in health. The rainbow model developed by Dahlgren and Whitehead [3] has been most widely used. They argue that the health of individuals who are endowed with age, sex and genetic factors are influenced by a number of layers that they diagrammatically present in the form of a rainbow (Figure 1): first, personal behaviour and ways of life that can damage or enhance health. These are influenced by social and community networks, which in turn, are contextualised by living and working conditions and access to facilities (including health and social care). The broader economic, cultural and environmental conditions in a society have a bearing on all the other layers below. This model, alongside others such as the one formulated by Brunner and Marmot [4] who include early life and cultural factors into the pathways to health, are primarily geared to an analysis of macro social factors. Further models can be added to the above approaches. These include the theoretical contribution of Bartley [5] who draws attention to further influences such as the physical environment of home, neighbourhood and workplace, and people's standard of living; the work on cumulative disadvantage through the life course by Davey Smith and Kuh, [6] and how these may be exacerbated through living in particular places [7].

The individual-oriented and the structural approach to health and illness are increasingly seen as to provide only partial answers to how to understand people's health status, and a body of work has emerged in the last few years that offers a new theoretical direction. Frohlich et.al. [8] argue that the missing question centres around understanding the relationship between agency (the ability for people to deploy a range of causal powers), practices (the activities that make and transform the world we live in) and social structure (the rules and resources in society). They critique the behaviourist interpretation of lifestyles and instead propose to define them as patterns and ways of living or as behaviours and their interactions with cultural, social and psycho-social factors. This leads them to conclude that it is important to recognise that the context (or structure) acts on individuals, but in turn individuals are re-creating the conditions that make this context possible (agency). We have, therefore, sought to explore aspects of agency and structure alongside Dahlgren and Whitehead's rainbow model in order to reveal a more nuanced understanding of the determinants of health in relation to knee pain and disability in older adults. Our study uses the model as a point of departure for a conversation about people's own health, their practices - in particular those intended to prevent ill-health, knee pain and disability - and the context which might shape their decisions.

The rainbow model is particularly suited to the study of knee pain and disability for a number of reasons. Firstly, the main determinants of health it outlines (for example exercise, diet, education, health services) are also core treatments for the effective care and management of knee osteoarthritis (OA) [9]. However, there is evidence of the under use of exercise, weight loss and written information in knee pain patients [10]. Exploring these determinants may therefore shed light on reasons for this. Secondly, items on the rainbow (for example work conditions and diet) reflect opportunities for prevention activity that have been identified in epidemiological studies and randomised clinical trials [e.g. [11,12]] but which have not been explored in detail at the individual level. Thirdly, the new Musculoskeletal Services Framework recommends integrated care pathways which focus efforts at self help and prevention [13]. Despite this, there is a lack of research into views of prevention for knee pain, and therefore a lack of knowledge on how such integrated pathways could be operationalised, or on what prevention activity would be acceptable or relevant to people in the context of their everyday lives.

## Methods

There were three aims of this study. First, to investigate people's perceptions of prevention of knee pain and disability and the factors that enhance or restrict prevention activity. Second, to use Dahlgren and Whitehead's rainbow model to explore in more detail a range of social determinants and how people make sense of these in relation to prevention of knee pain and disability and their health in general. Third, to consider the findings in terms of agency and structure.

Semi-structured interviews were undertaken with people from an existing knee pain cohort (KNEST study) [14,15]. In 2000, a questionnaire was mailed to 8995 adults aged 50 and over registered with three GP practices. 6792 people responded to the survey (77%) of which 5784 were still registered with the GP 3 years later when a follow up survey was administered. 58% of responders to the follow-up survey gave consent to further contact. Our sample was drawn from people who had given this consent.

The interview started with exploring people's general health, followed by a discussion of the influencing factors using a pictorial representation of the rainbow model as a reference point. While the second part of the interviews centred around the model, the semi-structured format allowed people the freedom to discuss all or just a section of the model, and to elaborate as they saw fit.

A topic guide for the interviews was developed and discussed at a Knee Pain Forum [16]. This group contained patients, health and social care professionals and representatives from other community groups. The Forum guided the research team on if and how to use the rainbow picture within a qualitative interview and recommended a pilot study. We therefore undertook a small pilot study with eight people. The aim of the pilot study was to test out using the rainbow model in a qualitative interview. The results of this are reported elsewhere [16]. The main study invited 180 people by letter to participate. The sample was selected purposively from the cohort study. We aimed to interview people with recent pain, resolved pain, or who were symptom free but who had at least two risk factors for knee pain (e.g. a previous knee injury, had a body mass index of 25 or over and classified as overweight or obese, or had a previous manual job). We also selected an equal number or men and women and sampled to ensure people were invited from across three age groups (50-64 years, 65-74 years and 75 years and over). People could either return a reply slip or telephone the researchers if they wanted to take part. Twenty people volunteered to take part. This seems a low uptake, but part of the reason for this may have been that people who were pain free, or who had resolved pain did not feel that the study was relevant to them. This paper reports on the total sample of 28 patient interviews (pilot and main study). As the interview method did not significantly change for the main interviews we felt that it was appropriate to combine the pilot and main datasets, particularly as we had low uptake in the resolved and pain free groups.

This final study sample included eight women and twenty men, aged between 53-86 years and is outlined in Table 1. Thirteen of the participants had current knee pain, two had resolved pain and thirteen were pain free. In relation to social class status [17] ten participants were classified as being, or had been, in professional or managerial occupations, eight intermediate and ten manual or routine occupations. Each interview lasted between 40 and 90 minutes (the majority were carried out by TO), were tape recorded and fully transcribed. Field notes were written after each interview. Common information and consent procedures were adopted in line with the approval by the Local Research Ethics Committee.

**Table 1:** Profile of Interviewees

ID	Gender	Age	Knee Pain Status
539	Male	58	Resolved pain
668	Female	61	Pain
669	Male	64	Pain
957	Female	64	No Pain
968	Female	59	No Pain
1148	Male	63	Pain
1417	Male	75	Pain
1633	Female	70	Pain
2241	Male	86	No pain
2390	Male	59	Pain
2411	Male	61	No Pain
3641	Male	78	No Pain
4237	Female	66	No Pain
4477	Male	69	Pain
4575	Male	60	Pain
4713	Female	63	Pain
5217	Male	72	Pain
5228	Male	62	Pain
5248	Female	79	Pain
5300	Male	68	Pain
5456	Male	68	No Pain
5969	Male	53	Resolved Pain
6028	Male	69	No Pain
6150	Male	62	No Pain
6545	Male	66	No Pain
6598	Female	61	No Pain
6894	Male	59	No pain
6900	Male	69	No Pain

**Figure 2:**
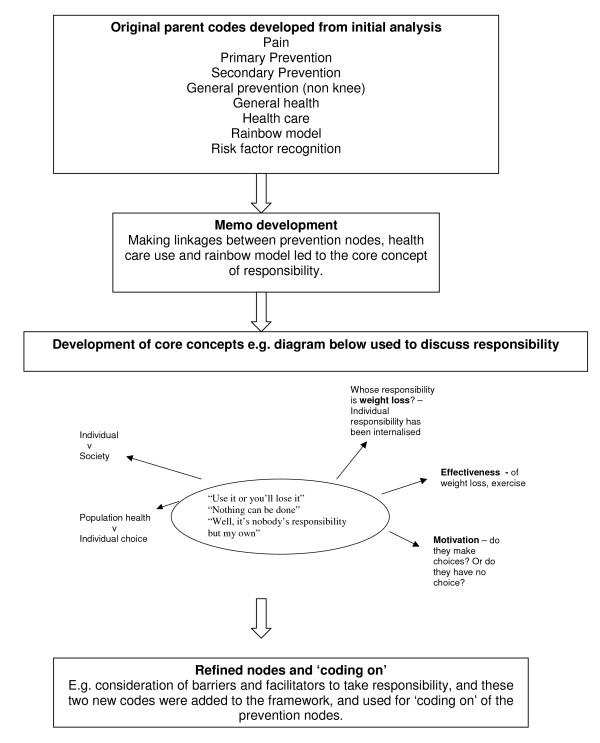
**Development of coding framework**.

The three researchers read the first three transcripts independently from each other and a coding framework was then developed through an iterative process and through discussion and comparison of data. Overall, there were few anomalies in interpretation when devising the coding frame and this is probably due to the similar backgrounds of the three researchers. The framework was used to code the remaining interviews. Qualitative computer software package NVivo 2 was used to manage the data. A thematic analysis approach was adopted. The codes were grouped in relation to the Rainbow model, and team analysis of the coding groups allowed relationships between the elements of the model to be drawn out that could then be ordered into themes. TO and CJ also wrote memos in order to develop ideas about the data and codes. This is an important process as it enables the researcher to commence data analysis early and capture comparisons and directions to pursue [18]. The main themes to emerge from the data were in relation to individual responsibility for health, barriers and facilitators to prevention activity and social context. Figure 2 outlines the development of the coding framework and the integration of memos within this.

## Results

### Whose responsibility is it?

The interview started with asking people about their general health, followed by exploring their knee pain history. This then led to talking about the factors that contributed to their knee pain and their thoughts about preventing this, using the rainbow model as a guide. While nearly all respondents alluded to the issue of responsibility for maintaining health, eight people elaborated on this concept in greater depth. Each person emphasised that individuals are responsible for their own health using phrases such as:

"Well, it's nobody's responsibility but my own." (4575) or "I think you have to be prepared to help yourself." (5300)

These statements became more nuanced through considering the factors that allowed people to live responsibly, and professional advice was seen as important, or more broadly, organisations should take a pro-active approach to individuals:

"With the National Health, you know, the lessons I've learned is that, if it's a great expense for them to react to a condition that I've had, whereas it might have been cheaper had the resources been there to have taken the preventative." (6028)

and

"I suppose, in the first instance, it's the individual, but people, being individuals, are all different. So I think there should be some sort of 'gee-up' from authorities [...] somebody could come round from the [city] council or some gym or something. Just do a few basic exercises with residents." (6900)

The role of health and other statutory services with respect to prevention were explicitly recognised by these individuals: the NHS was seen as focusing on treating acute problems which may have been prevented if investments were aimed at prevention rather than cure. The importance of organisations outside of health was highlighted in the second quote where it was suggested that city councils could create the conditions that helped people to improve their health. As an extension of organisational facilitators the professionals working within health and social care were seen to be important in advising people on healthy lifestyles and who should act as educators and sources of information. At the same time, their influence was dependent on individual receptivity:

"I think it's got to come from within, hasn't it really? I mean, when we, you can listen to advice, but you've got to either put that advice into practice or be determined enough, in your own mind, that, you know, you will either lose the weight or stop smoking, or stop drinking, or whatever." (4713)

This person connected three factors: being given advice, accepting and implementing it, and maintaining new behaviour as dependent on motivation. He reinforced this with giving an example of individual will-power (resisting social pressure to smoke and drink) and returning to the theme of individual responsibility by concluding "the way you live your life is up to you". Having a sense of responsibility was considered to be learned behaviour, and people linked it directly to their social context such as coming from a "reasonably stable family background" (5228) or parenting which included encouraging children to eat healthily and take exercise (957). A broader role for education was also mentioned with teaching children how to look after their own health (5969).

Two people went further and discussed the role of the state in the face of people abdicating responsibility for themselves. One person gave the example of his son, an ambulance driver, who told him about being called out for trivial things:

"So, I'm aware that a lot of people, you know, feel that or choose to take the attitude "well, I'd rather somebody else take the responsibility for what I should be doing myself", but I just don't happen to believe that." (5228)

However, he realised that there will always be people who will not take responsibility for themselves and felt the community and the state will need to provide protection or coercion:

"Ought the state be sort of saying that you don't do this or you do something else and stopping people from doing things?" (5228)

Individual responsibility for health emerged as a strong theme, but structural factors were considered to affect individual behaviours. Thus, Blaxter's [19] finding that people tend to see healthy lifestyles and personal character and determination as key factors in maintenance of health is supported by the explanations given in our study. At the same time the argument by Hodgins et.al. [20] is reflected in the responses that argue that a sense of responsibility is shaped by social influences such as education, family context and pro-active interventions by public sector organisations, thus connecting agency and structure in specific ways. Taking responsibility is, therefore, not purely dependent on an individual's strength of character, but is fostered and supported over one's lifetime by factors outside of oneself. By interpreting those influences, individuals in turn, shape and reinforce the context within which they live, for example, by outlining how they should be supported by organisations and the state.

### Barriers and facilitators to preventing knee pain and disability

Frohlich et.al. [8] developed the concept of collective lifestyles as "an expression of a shared way of relating and acting in a given environment" (p.791), but that can be observed through individuals' lifestyle practices. They further add a recursive aspect by stating that individuals are influenced by the context within which they live, but individuals also create and recreate the conditions that maintain structures. This line of thinking is helpful in making sense of how the people in our study reflected on the structural aspects of the rainbow model, and in particular, how they saw barriers and facilitators to health maintenance.

The majority of participants highlighted the importance of exercise to maintain musculoskeletal health. One person aptly termed it "use it or lose it", but this awareness was clearly tempered by contextual factors. The cultural perception of the word exercise tended to be equated with gyms, classes and specific regimes. A number of barriers were associated with this conceptualisation: first, gyms are for young people dressed in tight fitting clothes. One lady said that her husband did not want to entertain the idea of this type of exercise:

"He won't go to the gym. It's he doesn't want to go where the young 'dudes' are, you know, that's daft to him." (968)

Second, the issue of cost to either the individual or to the state when providing free exercise classes. The latter was illustrated by someone who saw the benefit of 'exercise on prescription' schemes, but that these could not be sustained indefinitely:

"After three months, you know, they've had the chance, you know. They can't keep saying: "well, I want to do, I want another three for free", you know. It can't go on forever. It's got to stop somewhere because it's going to cost too much." (5969)

This dilemma was particularly interesting as the collective understanding of the health benefits of exercise was supported by the state, but this support was time limited in the expectation that individuals would take over themselves. Two barriers might play a part: actual financial costs being judged by individuals as too high, or an expectation that it should be the state's rather than individual responsibility. Obviously, this participant felt that people should be given the opportunity to try out gyms, but that they should continue themselves without state support. The boundary between enacting a collective lifestyle with collective or individual resources has become fuzzy in this instance, and made it difficult to judge when and at what level costs would become a barrier.

The culture of work was mentioned by some people as a barrier, such as office jobs leading to a sedentary lifestyle, or the fact that work routines appeared to dominate everyday life:

"I mean, even at your age, you could look at some of the activities going on 'round you and you could think "well, I wished I could do, I wished I was doing that or not could do it, I wished I was doing it. Then you look at all your commitments. You say, I haven't got time." (6545)

This person saw work commitments determining how time was spent, and thereby reinforced the culture of work and its impact on people's choices. Yet, another person gave an example of how people could become empowered to challenge this:

"There's still people with busy lifestyles who build some exercise into it. Erm, like my daughter and her partner, her husband, go both out to work full time, but they do exercise. But they have a routine, they build it into their life." (5969)

Without explicitly referring to will power, the elements of personal choice and determination were implicit in this account, and the impact that individual behaviour might make on the environment was mentioned when concluding: "I think that people are slowly being made aware of it."

An important barrier to prevention was the actual image of knee pain itself as most people thought that "nothing could be done". As highlighted in previous studies [21,22] this perception was associated with ageing and inevitable 'wear and tear' of joints. We will not repeat the arguments from the literature here, but suffice with stating that in our sample considering knee pain as part and parcel of becoming older was internalised, and thus created a barrier to prevention. The fact that the health service and health professionals tended not to offer active interventions (apart from knee replacements in extreme cases) could be perceived as a structural (organisational) problem.

When broadening out the concept of gym-based exercise to staying active in a variety of ways, more than half of the participants mentioned the benefits of swimming, walking, cycling or dancing. The main facilitating factor in taking up activities appeared to be the social aspect:

"We do a lot of dancing at parties and that sort of thing, you know. I mean, well, I don't dance particularly well, but I hold this girl while she does, you know, what I mean. I'm daft and that, like we do a lot, I do enjoy that, you know and she does, because she's a dancer." (6900)

This gentleman emphasised his enjoyment of dancing, and the pleasure he derived from the dance partnership. The contacts with others in the parties made the activity of dancing a shared experience. Similarly, the lady who joined in with an over-60s gym club emphasised the social interaction:

"They're all the same as me. They're overweight and they've all got health problems [...]. We giggle, yeah, we giggle, you know. You know, sort of, I don't know, you even, we just say, you know: "How's your leg today?" "Oh, it's been awful this weekend, how's yours?" (968)

The important additional factor for her was the fact that she identified with her fellow club members in terms of health and mobility. They could ask each other about problems, be supportive and at the same time downplay issues through the use of humour. Thus, participation was stimulated by a combination of factors, and not just by the knowledge that activity would help with reducing pain and disability. The contribution of social networks, trust and support in maintaining health and well-being have been highlighted in the literature on social capital [23-25]. Similar results [26] have been documented in relation to coping with pain and the above accounts reflect these findings.

A further important facilitator was the issue whether activity could be integrated within everyday life, such as using stairs rather than lifts, walking instead of taking the car or the daily walk with the dog:

"That's what my specialist once said to me when I went. He said: "Have you got a dog, Mr.S?" I said "yes". He said: "Make sure he gets plenty of exercise." Ha, I thought, that's a nice way of putting it." (6150)

The facilitating factors clearly underlined the importance of context, and that taking up and maintaining an active lifestyle was dependent on social networks, access to facilities (for walking, cycling etc) and finally whether health policy supported healthy behaviours:

"Smoking has changed. Now, what's the difference between allowing, or taking action, of advertising and everything else to do with smoking, and yet, on the other hand the same government will then extend the licensing laws and allow alcohol to be drunk 24 hours a day." (5228)

The participants in this study demonstrated that the contextual opportunities shaped their ability to put into practice their knowledge about health enhancing behaviours, in particular when the benefits were wider than pure physical health and encompassed psychological and social well-being. Thus, it was clear that they connected the different levels of the rainbow model and perceived their own decision-making as embedded within all the layers. In this way the model made sense as an explanatory and holistic model, even though in terms of action people emphasised individual responsibility and behaviour.

## Discussion

The formulation of the rainbow model [3] was based upon the understanding that biological factors, social and physical environments, personal lifestyles, services and policy all influence the health status of individuals. These various factors are interrelated in complex ways, and determine the patterning of health inequalities. While this model has shaped public health thinking and policy with regard to populations, it has not been applied at the individual level. The purpose of our study was to use the model to help participants make sense of their own lives, and in particular when considering prevention of knee pain and disability.

The participants considered the model as a common-sense framework for locating their personal life within a wider social, economic and policy context and made reference to a range of factors that have shaped their thinking about health and prevention. It appeared that the majority of people considered individual responsibility for health maintenance as the key, but in the detailed discussions about the interaction between the layers within the model a more nuanced approach emerged. The relationship between agency, practices and structure [8] was drawn by a number of participants when they explained how their power to act was fed by feeling confident as a result of their upbringing or educational achievement. Their beliefs about appropriate health behaviour could be put into practice when stimulated or supported by professional advice, structural opportunities such as access and financial resources or in some cases by policy initiatives. While the words being used appeared to be individual-orientated - such as 'determined', 'help yourself' or 'it is down to you' - they were not purely connected with moral fibre as defined in Blaxter's study [19]. In particular, when considering barriers and facilitators to preventive action the influence of social structure on individuals' interpretation of their everyday reality reflected how they took into account factors beyond physical health and talked about well-being with regards to social and psychological aspects over the life course. This supports the notion that Williams [10] proposes, namely to develop a sociological and historical analysis of people and the places within which they live, and use this to contextualise patterns of health behaviour and outcomes. In our study people clearly engaged in such a fine-grained approach to their health behaviour by referring to their past, present and expectations of the future as embedded within their social context and the opportunities (or lack of) that these afforded.

## Conclusion

The specific relevance of the rainbow model to knee pain relates to the NICE guidelines emphasising individual behaviours and self care, the evidence from epidemiology that identifies risk factors and opportunities for prevention, and the findings from qualitative studies that provide more complex and detailed understanding of living with knee pain.

We conclude that the model does have relevance at the individual level and can help formulate more dynamic and contextualised approaches that resonate with individuals' own interpretation of their lives, health and prospects for deliberate action that go beyond the duality of agency and structure. This is particularly important given the increased emphasis on partnership between patients and health care professionals, whereby a better understanding of the life-world of patients allows for consultations to be responsive, targeted and more likely to lead to improved outcomes.

## Competing interests

The authors declare that they have no competing interests.

## Authors' contributions

CJ and BNO conceived the design of the study. TO coordinated the study and undertook the qualitative interviews. All authors contributed to interpretation of data and to the development of this manuscript. All authors read and approved the final manuscript.

## Pre-publication history

The pre-publication history for this paper can be accessed here:

http://www.biomedcentral.com/1471-2458/10/148/prepub
